# The Vulnerability Scan, a Web Tool to Increase Institutional Biosecurity Resilience

**DOI:** 10.3389/fpubh.2019.00047

**Published:** 2019-03-12

**Authors:** Stephanie E. Meulenbelt, Mark W. J. van Passel, Arnout de Bruin, Linda M. van den Berg, Mirjam M. Schaap, Saskia A. Rutjes, André J. Jacobi, Marja C. Agterberg, Carin de Hoog, Gijsbert van Willigen, Evelien Kampert, Jan H. J. Heres, Ruud van den Berg, Harold H. J. L. van den Berg, Diederik A. Bleijs

**Affiliations:** ^1^Netherlands Biosecurity Office, National Institute for Public Health and the Environment, Bilthoven, Netherlands; ^2^Centre for Zoonoses and Environmental Microbiology, National Institute for Public Health and the Environment, Bilthoven, Netherlands; ^3^Leiden University Medical Center, Leiden, Netherlands; ^4^National Institute for Public Health and the Environment, Bilthoven, Netherlands; ^5^Netherlands Food and Consumer Product Safety Authority, Wageningen, Netherlands

**Keywords:** biosecurity, biosafety, vulnerability, web tool, Gap analysis method, risk assessment, awareness raising

## Abstract

The importance of vigilance within organizations working with high-risk biological material receives increasing attention. However, an in-depth and comprehensive tool, dedicated to increase awareness of potential risks and to assess an organization's current biosecurity vulnerabilities, has not been available yet. We developed the “Biosecurity Vulnerability Scan,” a web tool that identifies biosecurity gaps in an organization based on eight biosecurity pillars of good practice. Although the tool aims primarily to assist biosafety and biosecurity officers, it can also be useful to researchers working with dangerous pathogens, their principal investigators, management, or those responsible for security issues in the life sciences. Results are only stored locally and are provided in an “overview report,” which includes information on relevant risks and control measures. This can support well-substantiated decision-making on strengthening biosecurity measures within a specific organization. With this article, we aim to support institutes to increase their overall security resilience and to improve institutional biosecurity in particular by providing practical recommendations. The Biosecurity Vulnerability Scan is available at www.biosecurityvulnerabilityscan.nl

## Introduction

Many organizations, including hospitals, biotechnology companies, and universities, work with biological material. In case of accidents, theft or misuse, some of these biological materials can pose a risk to human, plant or animal health. Therefore, organizations have a responsibility to take measures to prevent such materials from causing harm. It is thereby important to pay attention not only to biosafety, which focuses on preventing unintentional release of hazardous biological materials, but also to biosecurity aspects, where the aim is to prevent misuse by, for example, intentional release of such materials (1). Furthermore, laboratory biosecurity measures intend to protect society not only by preventing the misuse of biological agents, but also to prevent misuse of related knowledge and technologies.

The importance of taking risk-mitigating measures within organizations handling high-risk biological materials has received increased attention. Not in the least since several (preventable) incidents took place in the past years. For example, while doing an inventory of their lab in preparation for a move, the US Food and Drug Administration discovered vials labeled with variola (smallpox) in 2014, a virus which should only be available at the CDC in Atlanta, USA and the VECTOR institute in Novosibirsk, Russia (2). In that same year, additional incidents were reported, including the mishandling of *Bacillus anthracis* spores, and the shipment of a low pathogenic influenza virus sample contaminated with a highly pathogenic Influenza strain (3). These examples highlight several aspects that can go wrong when working with pathogenic organisms, including issues with accountability for materials and transport security. Fortunately, these incidents have been properly notified, and now serve as examples from which the wider community can learn.

In addition to the importance of taking appropriate biosecurity measures, there is the importance of a risk assessment covering dual use aspects of research in the life sciences. This assessment should weigh the risks and benefits of dual use research of concern, which is research that is beneficial for society, but the generated knowledge, technologies or pathogens could potentially also be misused as biological weapon (4). Recently, Noyce et al. published a study on the synthesis of a viable and infectious horsepox virus, a virus related to smallpox (5). The journal's publisher stated that a dedicated committee had concluded that the benefits of this study outweighed the risk of misuse (6), but the publication of the methods for the synthesis of infectious viruses raised concerns with health security experts.

In order to minimize biosafety and biosecurity incidents and risks in the future, a range of awareness raising activities, education programmes as well as (e-learning) tools have been developed and implemented recently, often freely available. Examples are the United Nations Food and Agriculture Organization Biosecurity Toolkit manual (7), Sandia National Laboratories Biorisk Assessment Models (BioRAM) (8), the Danish Handbook on Biosecurity (9), the textbook “Preventing Biological Threats” (10), a biosecurity survey carried out in Kenya using lab visits with a standardized questionnaire (11), the CEN Workshop Agreement on Laboratory Biorisk Management (1), the Netherlands Code of Conduct for Biosecurity (12), and the Biosecurity Self-scan Toolkit developed by the Netherlands Biosecurity Office (13). However, these initiatives are rather abstract or focus on particular regions or countries, branches or specific aspects of biosecurity.

Through field consultations, the Netherlands Biosecurity Office noticed the need for a more comprehensive assessment tool, containing nuance and detail in questions. The Netherlands Biosecurity Office is the national information center for the Government of the Netherlands and for organizations that work with high-risk biological material. It aims at increasing biosecurity awareness and decreasing biosecurity vulnerabilities in organizations in the Netherlands and abroad (13). It does so by disseminating knowledge and information about biosecurity, giving workshops, lectures and developing web tools. The findings of the field consultations are used to develop the Vulnerability Scan: a comprehensive, web-based tool, usable by a broad range of organizations that work with high-risk biological material.

This article describes the development process and the set-up of the Vulnerability Scan as well as how practitioners can use it to raise awareness of institutional biosecurity and decrease biosecurity vulnerabilities within their respective organizations. We aim to support institutes to increase their security resilience in general and improve their institutional biosecurity in particular. Since our tool includes questions that help identify potential biosecurity weaknesses within an organization, real-life scenarios and practical implementation recommendations, we hope the article inspires and encourages practitioners to use our tool in order to do so.

## Development

The Vulnerability Scan has been developed in response to the identified need in the field for a more comprehensive and detailed (self-)assessment tool. A panel of biosafety and biosecurity experts as well as end-users were consulted throughout subsequent phases of the development process. The consultations took place as a series of face-to-face discussions as well as consultations per e-mail. Particular subject-matter experts were also consulted, e.g., cybersecurity professionals for their expertise on information security. This allowed for the development of a tool that primarily provides guidance to biosafety and biosecurity experts, but can also prove useful to researchers, principal investigators, and security experts of institutions active in the life sciences, such as hospitals, research institutes, universities, and biotech companies.

The Netherlands Biosecurity Office developed the first draft of the Vulnerability Scan in close cooperation with several and biosafety and biosecurity experts. This group convened in several meetings to compose the tool and ascertain applicability for the intended users. Starting point for the development of the Vulnerability Scan was the Self-scan Toolkit. This Toolkit includes eight biosecurity pillars, which are also taken as a basis for the Vulnerability Scan. However, rather than including 10 “yes” or “no” questions per pillar, multiple-choice questions have been built-in the Vulnerability Scan to provide a more nuanced view on biosecurity issues. In addition to the sets of multiple-choice questions, scenarios reflecting realistic situations and practical implementation recommendations were included. We want to challenge users to critically reflect upon the biosecurity level within their organization(s) by going through the questions and discussing the dilemmas provided in the real-life scenarios.

When the development team was confident on the content, it was processed into a usable online tool. The first version was thereby ready for testing. For this beta-test, 40 experts were selected, including biosafety officers, occupational health professionals and safety experts with expertise in human, animal or plant high-risk pathogens. All experts received a standardized questionnaire containing queries on overall impression of the tool, user friendliness, whether, or not expectations on content and goals were met, use of language, whether, or not they missed certain aspects, etc. Furthermore, each individual expert was asked to critically assess two pillars: one that was directly within their area of expertise and a second one that inspired their interest. The development team carefully analyzed the experts' input to improve the content of the tool. After this input had been processed, the Vulnerability Scan was deemed ready to use and could be officially launched in November 2017.

**Figure 1:**
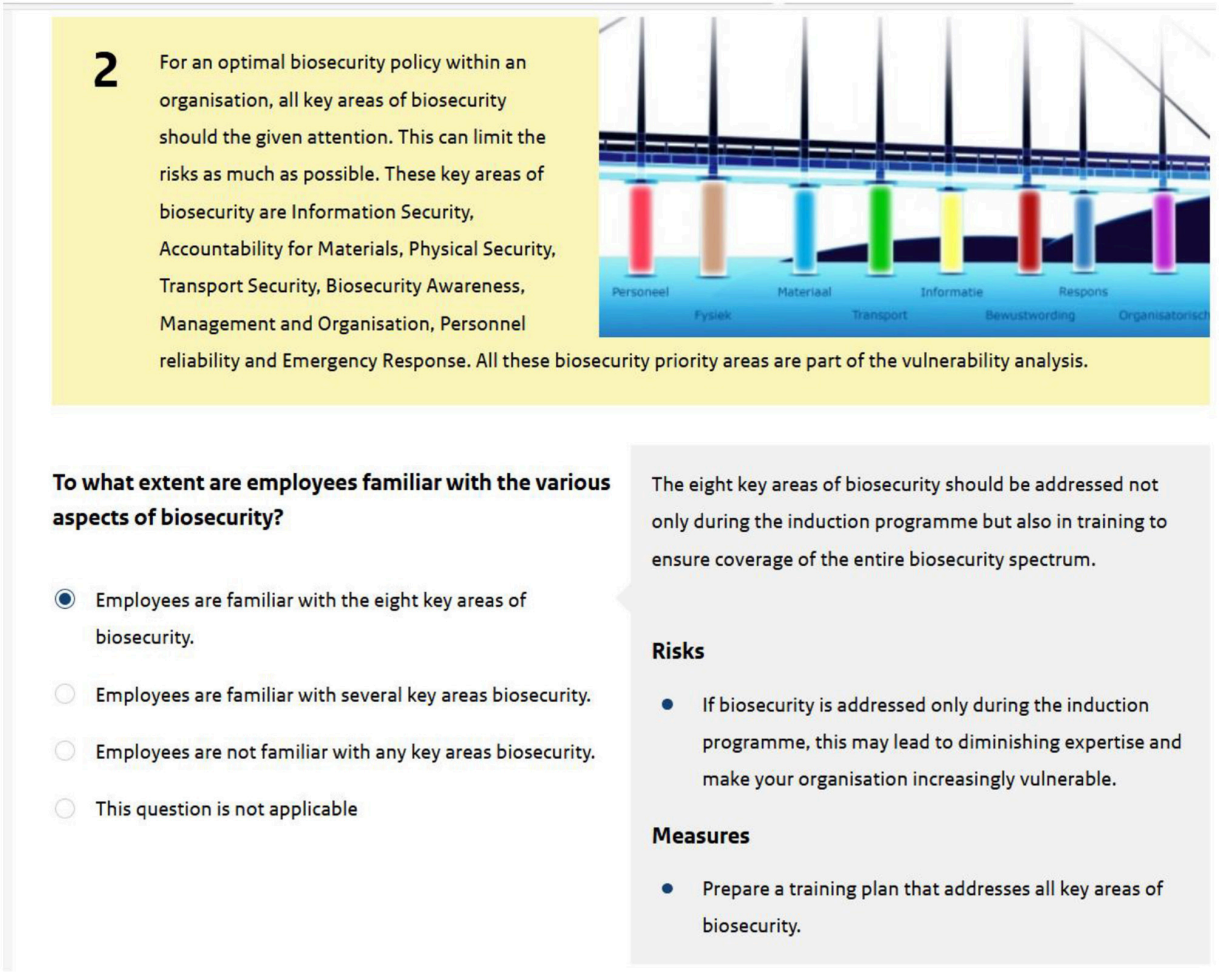
Screenshot of the vulnerability scan. Taking question two of the pillar Biosecurity Awareness as an example, it shows both possible answers, and the structure in which answers are provided.

The current version of the Vulnerability Scan consists of eight pillars of good practice representing all key areas of biosecurity: Biosecurity awareness; Personnel liability; Information security; Management; Accountability for materials; Physical security; Emergency response; and Transport security. Each pillar contains a minimum of three questions with a number of possible answers as well as a set of risks and risk management strategies (Figure 1). Likewise, each pillar holds several different scenarios, accompanied with a short description and identification of possible risks. The scenarios should trigger questions if the depicted situation is recognizable, e.g., is the situation realistic; what are the vulnerabilities or possible consequences in this situation; and what control measures can be taken to prevent the scenario from occurring?

Since technological developments are expected to change the biosafety and biosecurity landscape, it remains important to continuously update the Vulnerability Scan. After its first testing in 2017, we received feedback from users, and we encourage users to continue to provide suggestions for improvement of the tool. For example, it could be very interesting to learn more about best practices in the field, which could be incorporated into (the multiple-choice questions of) the tool. Or, real-life (anonymized) scenarios could be added or adjusted on the basis of incidents that occurred within relevant institutes.

Finally, in order to properly assess the biosecurity vulnerabilities in one's organization, it is crucial that the user feels comfortable to answer the questions honestly. Therefore, the vulnerability scan is specifically designed to be anonymous. The results are not shared with the Biosecurity Office or other third parties, the results are stored locally on the user's computer as cookies, in order to continue previous sessions.

## Application

The aim of the Netherlands Biosecurity Office was to develop a tool that provides a thorough analysis of an organization's level of biosecurity and offers an organization a specific picture of the biosecurity state-of-the-art, including both strengths and especially vulnerabilities. Identifying vulnerabilities is an import step toward a biorisk assessment. Simultaneously, using this tool raises awareness on the eight pillars of good practice, representing all key areas of biosecurity (13). The Vulnerability Scan also allows institutional self-auditing, including monitoring the mending of biosecurity vulnerabilities over time. Considering that all pillars are a prerequisite for a sound biosecurity system, they are given in no specific order. A description of the pillars is given below.

### Biosecurity Awareness

Biosecurity awareness is of utmost importance to generate a safe and secure biosecurity culture within organizations. Employees should be aware of these risks in order to take corresponding measures. This enables employees to identify hazards, remain vigilant, and comply with existing rules. Biosecurity awareness enables employees to assess potential dual-use risks, recognize abnormal situations and hold colleagues accountable for their behavior. For example, this pillar includes a scenario on dual use aspects of research in the life sciences related to an experiment that results in a dramatic increase in virulence of an otherwise non-pathogenic strain. It intends to make the user aware that, although insights gained in such experiments may lead to better treatments of infectious diseases, the results could also be misused if they fall into the wrong hands. This pillar provides insight into the extent to which employees are aware of the eight biosecurity pillars of good practice, helps to bring these to the attention of the employees, and the importance of coherence between them. By being aware of the importance of biosecurity, both management and employees will ensure biorisk management.

### Personnel Reliability

Reliable, well-trained staff is vital for biosafety and biosecurity management within an organization. The policy of an organization regarding personnel reliability should include a selection procedure for new staff, including temporary employees, external employees, and maintenance staff. Security risks can be mitigated by implementing a thorough selection procedure and an appropriate background screening for employees handling confidential data or high-risk pathogens. In addition to access control measures, conscious, and aware employees contribute to a secure situation. This pillar of good practice provides insight into selection procedures for personnel, types of background screening for personnel, security risks concerning behavioral changes and working outside regular hours.

### Information Security

In order to set-up this pillar, several information security managers were consulted and three key concepts regarding information were identified and included into the tool: Availability, Integrity and Confidentiality (AIC). “Availability” safeguards the security of systems and manages the risks of mistakes, malfunctions, and incidents. “Integrity” (or reliability) ensures that data are correct and up-to-date. “Confidentiality” (or exclusivity) ensures that only authorized personnel have access to systems and data. Information security overlaps with other pillars such as physical security and awareness. This pillar provides insight into information security management systems, availability, and continuity of such systems, integrity, and reliability of information, and their confidentiality and exclusivity.

### Management

Management, such as directors and principal investigators, plays an important role in preparing and implementing policy on key areas of biosecurity. If management is aware of the advantages of an effective biosecurity policy, the implementation and monitoring of biosecurity measures can be facilitated. This includes formulating procedures and regulations, and assigning roles and responsibilities to staff with respect to biosecurity or biorisk management. For example, one of the questions that practitioners find in the tool under this pillar is: How is management involved in biosecurity? Answer possibilities range from “highly engaged” to “unaware of biosecurity aspects,” each with matching risks and measures that can be taken to mitigate them.

### Accountability for Materials

The characteristics of the high-risk material are important for the biosecurity risks associated with handling and storing of high-risk pathogens. Route of transmission, pathogenicity, and availability of the material in combination with research technologies used and the knowledge generated might increase the risks of potential misuse of the material. To mitigate biosecurity risks the organization should be accountable for the material and store high-risk material in a controlled and secure environment, including: identification, registration and management of material. This pillar provides insight into the assignment of responsibilities for registration and management of high-risk materials, awareness of the difference between biosafety (working safely with high-risk materials) and biosecurity (securing high-risk materials), specifically with respect to localization and identification and the dual use aspects.

### Physical Security

Protecting, authorizing, and controlling access to vital areas using several security layers prevents unauthorized persons to gain access to high-risk materials. Therefore, the pillar “Physical security” focuses on access authorization and control, and other physical security measures. During the development phase, consulted physical security experts suggested to include the concept of Defense-in-Depth (layered or fragmented security), which is amongst others a concept that is used in nuclear and chemical industries. It includes a combination of architectural, technical and organizational aspects and can provide valuable insights to organizations working with high-risk biological material as well. In addition to providing insight into several aspects of layered or fragmented security, the pillar Physical security also addresses types of physical measures and security systems, authorization, and control of access and employee awareness and alertness.

### Emergency Response

Emergency (response) plans are essential and provide guidance to an emergency response service of an organization. A response plan describes the course of action and coordination of staff and services involved in emergency response activities within the organization, but should include collaboration aspects with external emergency response services as well. Emergency response plans contribute to prevention and control of incidents, accidents and emergencies with high-risk pathogens. Appropriate emergency planning and preparations facilitates immediate action upon inadvertent exposure of employees to fire, biological agents, or monitor vital areas during an incident.

### Transport Security

Transport security includes measures and procedures for the protection of high-risk pathogens during transport. During transport of high-risk material, neither the shipper nor the receiver has control over the material. This pillar is intended to identify the vulnerabilities within the entire transport process, including packaging and sending and receiving high-risk material. For transport of biological materials (such as infectious microorganisms, genetically modified organisms (GMOs), diagnostic samples, and hospital waste, including toxins) by road, water, rail and air, national and international laws and regulations are in place. Moreover, additional legislation could be applicable, for example, when pathogens are listed on an EU export control list of the dual-use regulation, US select agents list or the Australia Group.

For each of the eight pillars of good practice, a multiple-choice questionnaire and a number of scenarios have been developed. The possible answers are supported by an explanation, followed by a number of control measures one's organization may consider to mitigate biosecurity risks. In addition, the provided scenarios may offer additional insight into potential (bio)security risks within an institute, or could be used in table-top exercises. After completing all (or parts) of the scan, an overview report is generated, which provides insight in vulnerabilities and includes complementary information on associated risks and control measures. This report also includes suggestions and good practices one may want to consider to further improve the organization's biosecurity level. For convenience, an online user manual is provided at the homepage of the Vulnerability Scan.

Since the Vulnerability Scan assesses a number of different aspects within an organization, it is advantageous to complete the questions in the tool consulting several representatives of an institute. It is encouraged that the user assesses all relevant sections of the Vulnerability Scan, but it remains possible to focus only on a single, or several, pillars. These biosecurity gaps can subsequently be used to increase institutional biosecurity resilience, for example in conjunction with the WHO Laboratory biosecurity guidance (14).

In conclusion, the Vulnerability Scan enables the assessment of the current biosecurity level and identification of vulnerabilities within an organization based on eight pillars of biosecurity. The results are provided in a final report, which includes complementary information on associated risks and mitigating measures, supporting well-substantiated decision making on strengthening biosecurity measures within your organization. The web tool is freely available at www.biosecurityvulnerabilityscan.nl

## Disclaimer

Using the Vulnerability Scan is anonymous; data are stored locally on your computer only. This way, the vulnerability analysis can be completed at another time. Stored data can be deleted by pressing on the button “delete data” on the homepage. No rights can be derived from using this tool and the Biosecurity Office is not responsible for the outcome and results of the vulnerability scan. The tool identifies vulnerabilities and aims at increasing institutional biosecurity resilience and provides input for a risk assessment. However, it is no substitute for a biorisk assessment and the organization remains responsible for conducting risk assessment. For questions, remarks, or additional information or suggestions on the web tool, please contact the Netherlands Biosecurity Office at biosecurity@rivm.nl.

## Author Contributions

SM, MvP, AdB, LvdB, MS, SR, AJ, MA, CdH, HvdB, and DB contributed to conception and design of the web tool. GvW, EK, JH, and RvdB revised it critically for content. SM and MvP wrote the manuscript. All authors contributed to manuscript revision, and read and approved the submitted version.

### Conflict of Interest Statement

The authors declare that the research was conducted in the absence of any commercial or financial relationships that could be construed as a potential conflict of interest.
